# Genetic variants and their interactions in disease risk prediction – machine learning and network perspectives

**DOI:** 10.1186/1756-0381-6-5

**Published:** 2013-03-01

**Authors:** Sebastian Okser, Tapio Pahikkala, Tero Aittokallio

**Affiliations:** 1 Department of Information Technology, University of Turku, Turku, Finland; 2 Turku Centre for Computer Science (TUCS), Turku, Finland; 3 Institute for Molecular Medicine Finland (FIMM), University of Helsinki, Helsinki, Finland

## Abstract

A central challenge in systems biology and medical genetics is to understand how interactions among genetic loci contribute to complex phenotypic traits and human diseases. While most studies have so far relied on statistical modeling and association testing procedures, machine learning and predictive modeling approaches are increasingly being applied to mining genotype-phenotype relationships, also among those associations that do not necessarily meet statistical significance at the level of individual variants, yet still contributing to the combined predictive power at the level of variant panels. Network-based analysis of genetic variants and their interaction partners is another emerging trend by which to explore how sub-network level features contribute to complex disease processes and related phenotypes. In this review, we describe the basic concepts and algorithms behind machine learning-based genetic feature selection approaches, their potential benefits and limitations in genome-wide setting, and how physical or genetic interaction networks could be used as *a priori* information for providing improved predictive power and mechanistic insights into the disease networks. These developments are geared toward explaining a part of the missing heritability, and when combined with individual genomic profiling, such systems medicine approaches may also provide a principled means for tailoring personalized treatment strategies in the future.

## Introduction

Most disease phenotypes are genetically complex, with contributions from combinations of genetic variation in different loci. A major challenge of medical genetics is to determine a set of genetic markers, which when combined together with conventional risk factors could be used in predicting an individual's susceptibility to developing various complex disorders. The recent advances and wide availability of genetic technologies, such as those based on genome-wide association (GWA) and next-generation sequencing (NGS), have allowed for the in-depth analysis of the variation contained in the human genome. In particular, these technologies are enabling the investigation of the genetic architecture of complex diseases, with the aim of constructing more accurate disease risk prediction models that would eventually facilitate effective approaches to personalized prevention and treatment alternatives for many diseases [1,2]. While GWA studies have successfully identified hundreds of genetic variants that are associated with complex human diseases and other traits [3-6], most variants identified so far using mainly statistical association testing approaches only capture a small portion of the heritability and even an aggregate of these effects is often not predictive enough for clinical utility, leaving open the question of what may explain the remaining or ***‘missing heritability’***[7]. Suggested explanations include, for instance, contributions from rare and structural variants, genotype–environment and gene-gene interactions and sample stratification, or simply that complex traits truly are affected by thousands of variants of small effect size [8,9]. The relative contributions of these and other factors remain poorly understood, which is hindering the development of improved models for disease risk assessment.

Given the multi-factorial nature of complex diseases, many authors have reiterated the concept of interactions among genetic loci, so-called ***epistatic interactions***, as one of the major factors contributing to the missing heritability [9,10]. Epistatic genetic interactions between or within genes are thought to be profoundly important in the development of many complex diseases, but these interactions are often beyond the reach of the conventional single-variant association testing procedures [11-14]. There exist also increasingly complex interactions between genetic variants and environmental factors that may contribute to the disease risk on an individualized basis. Consequently, it has been argued that we should move away from the traditional ’one variant at a time’ approach toward a more holistic, network-centric approaches, which take into account the complexity of the genotype-phenotype relationships characterized by multiple gene-gene and gene-environment interactions [15,16]. Although the conventional statistical significance testing procedures have successfully identified several susceptibility loci, it has become clear that many of the true disease associations may be much lower down on the ranked list of hits, compared to the top hits with the most statistical support [4,17,18]. Ignoring the potential risk variants in this ‘*gray zone’* of genetic information is likely to result in models that are missing an important proportion of the quantitative variation in heritability. Therefore, it may be that most of the heritability is hidden rather than missing, but has not previously been detected because the individual effects are too small to pass the stringent significance filters used in many studies, yet still having significant contribution to the predictive power at the level of variant or subject subsets, or when combined with non-genetic risk factors.

Here, we discuss how computational machine learning approaches can utilize hidden interactions among panels of the genetic and other risk factors, predictive of the individual disease risk by means of implementing genetic feature selection procedures and network-guided predictive models. In contrast to the conventional population-level association testing, which often detect only a few variants with statistical support beyond the genome-wide significance level (e.g. *p* < 10^-8^), machine learning algorithms place special emphasis on maximizing the predictive accuracy at the level of individual subjects. The goal of feature selection is to identify such a panel of genetic and other risk factors, which result in a model that optimally predicts the phenotypic response variables, either the class labels in case-control classification (e.g. disease *vs.* healthy), or quantitative phenotypes in regression problems (e.g. height prediction). While epistatic genetic interactions may easily end up being averaged out in statistical association models, machine learning-based predictive modeling can also take into account those individual effects that are dependent on interactions with other variants or environmental exposures, making these models convenient for developing predictive strategies for multi-factorial diseases. Indeed, it has been shown that single-locus *p*-value-based selection strategies for constructing prediction models may lead to sub-optimal prediction accuracies [17]. In another example, hundreds of genetic markers, many of which did not originally meet the genome-wide level of statistical significance, were combined into a predictive model of type 1 diabetes risk [18]. Even though diabetes is known to involve many biological pathways, the large number of variants required may partly be attributed also to the selection of variants based solely on their individual *p*-values, which does not take into account any gene-gene interactions.

While machine learning-based computational approaches may provide a convenient framework for making use of the whole spectrum of genetic information when predicting an individual’s risk of developing a disease, these developments are still in their very early stages. Implementation of highly scalable computational algorithms for genetic feature selection is a key for making these frameworks effective enough for mining data from current GWA studies, in which more than a million genetic variants are assayed in thousands of individuals, not to mention the emerging data from NGS studies, such as the 1000 Genomes project [19]. Recent improvements in constructing accurate and scalable machine learning-based predictive models will be discussed in Section 2. Another pressing problem inherent in every machine learning application is the challenge of how to evaluate the predictive capability of the constructed models, in order to avoid stating over-optimistic prediction results [20]. Model validation approaches are described in Section 3. One approach to reducing the massive search spaces and computational complexities is to use additional biological information in the model construction process. There are already several successful examples of how to make use of physical protein interaction networks when mining data from GWA studies in the search of, for instance, regulatory models [16], epistatic interactions [21], or disease genes [22]. In Section 4, we take the next step of network level analysis of genetic variants and review recent data mining solutions capable of systematically utilizing functional information from the interaction networks as *a priori* information when building disease prediction models. Finally, in Section 5, we will list some current challenges and possibilities as future directions toward improved understanding of individual predisposition to genetically complex diseases such as cancers.

## Selection of genetic risk factors for machine learning-based prediction models

Rather surprisingly, the use of machine learning method in the context of genome-wide data on genetic variants has yielded a relatively limited number of studies until the very recent years (for a systematic literature review, see [20]), compared to the large number of machine learning studies on other types of genomic datasets, especially genome-wide gene expression profiles. Further, the combination of predictive modeling and advanced feature selection algorithms have been implemented in an even more restricted set of studies, even though these have generally yielded quite positive results [15,23]. Indeed, many studies have demonstrated that the use of feature selection approaches are capable of improving the prediction results beyond that when the same model is implemented on features selected solely through prior knowledge of the disease or on those genetic variants which reach genome-wide statistical significance [18,23-25]. However, it is relatively challenging to extract the predictive signal from the high-dimensional datasets originating from GWA or NGS studies, due to a number of experimental and computational issues, many of which are different from those faced when using data from microarray gene expression profiling. Further, in order to construct accurate and reliable predictive models of complex phenotypes based on genome-wide profiles of genetic variants, it is essential to have an understanding of how to identify predictive features both individually and in groups of variant subsets, and how different feature selection approaches can deal with issues such as epistatic interactions and high-dimensional datasets [15]. Feature selection methods in machine learning can broadly be divided into filter, wrapper and embedded methods. This categorization is not strict, and each of the approaches has its own advantages and disadvantages which are, in turn, very problem dependent. Next, we briefly describe each feature selection category and consider some representative examples of each.

### Filter methods

Filter methods for genetic feature selection are the most common in GWA studies due to the simplicity of their implementation, low computational complexity, and the human interpretability of the results. In their simplest form, filter methods calculate a univariate test statistic separately for each genetic feature, and the features are then ranked based on the observed statistic values. The highest ranked features form the final set of selected features, on which a predictive model may be subsequently trained. The number of features to be selected is either decided in advance or determined by a pre-defined significance threshold for the test statistic. Several well-known statistical tests have been used in GWA studies, including the Fisher’s exact test and Armitage trend test [26-28], and an increasing number of statistical approaches are being developed for rare variants and the NGS data [29-31]. Since this feature selection approach requires only a single pass through the whole data, single-locus filters can be straightforwardly applied to even the largest genome sequencing datasets. Along with the multiple testing problem, the primary drawback of the single-locus filter methods is that they do not take account of the interactions between the features, which may lead to selection of both false positives, such as redundant loci, and false negatives due to epistasis interactions between or within loci [12,13,15]. More advanced filter methods can also select specific risk variant combinations that are associated with a disease risk. For instance, multifactor dimensionality reduction (MDR) is a non-parametric method that can detect statistically significant genetic interactions among two or more loci in the absence of any marginal effects, even in relatively small sample sizes [32]. While proved to be useful for association testing, however, it has been argued that the statistics being used to identify variants or their combinations, typically *p*-values for disease risk association, are perhaps not the most appropriate means for evaluating the predictive or clinical value of the genetic profiles [33].

### Wrapper methods

Wrapper methods consist of three components: a search algorithm for systematically traversing through the space of all possible feature subsets, a scoring function for evaluating the predictive accuracy of the feature subsets, and the learning algorithm around which the feature selection procedure is wrapped [34]. Since the size of the power set of the features grows exponentially with the number of genetic variants screened (say *n*), testing all the feature subsets (2^*n*^) is computationally infeasible (*n* is on the order of a million in a typical GWA study and much larger in NGS studies). Therefore, one must resort in practice to local search methods that do not guarantee finding the optimal subset but, nevertheless, usually lead to good local optima. For example, the greedy forward selection adds one feature at a time to the set of selected features after checking which of the remaining features would improve the value of the scoring function the most. Thus, the whole data set is traversed through once for each selected feature. To avoid getting trapped in poor local optima during the search in the complex and high-dimensional genetic landscapes, modified local search strategies can be utilized, including the backtracking option or several variations of evolutionary algorithms. The most popular scoring functions used with wrapper methods are the prediction error on the training set, a separate validation set, or cross-validation error. The feature selection can be in principle wrapped around any learning method, but it is beneficial if the method can be efficiently trained or if the already learned model can be efficiently updated. Indeed, for some learning methods, such as regularized least-squares (RLS), the search process can be considerably accelerated with computational short-cuts for scoring function evaluation [23]. These inbuilt short-cuts bring the methods closer to the next category of the selection methods, namely the embedded ones.

### Embedded methods

Embedded methods have the feature selection mechanism built into the training algorithm itself [35], that is, the predictive models they produce tend to depend only on a subset of the original features. Perhaps the most well-known embedded method is LASSO (least absolute shrinkage and selection operator), which is also recently being applied to a larger number of GWA studies [20,25,36-38]. While only a few machine learning approaches, in fact, allow for scaling-up to the genome-wide level, this has been made possible in LASSO by the recently developed model training algorithms, such as those based on the coordinate descent methods, which are computationally very efficient. The problem setup resembles the wrapper approach in the sense that there is an objective function for which one performs a stochastic search, such as cyclic or stochastic coordinate descent, in order to find a global optimum. Basically, the search algorithm goes through each feature at a time, and updates the corresponding coefficient in the linear model under construction. The objective function consists of a scoring metric such as the mean squared error (MSE) on the training data and a regularization term that favors sparse linear models, that is, it tends to push the search algorithm towards such models that have only a few nonzero parameters. Typically, coordinate descent passes through the whole data set only a couple of times before convergence, but the number of passes depends on the properties of the data, the desired sparsity level, and the other possible hyperparameters. Wrappers and embedded methods are known to have the ability to produce better results than filter methods in many applications [23,25,39], but if not implemented correctly, they can easily lead to the models failing to generalize beyond the training data, underscoring the importance of rigid evaluation of the prediction models.

## The importance of evaluation of the predictive models for complex phenotypes

**Figure 1:**
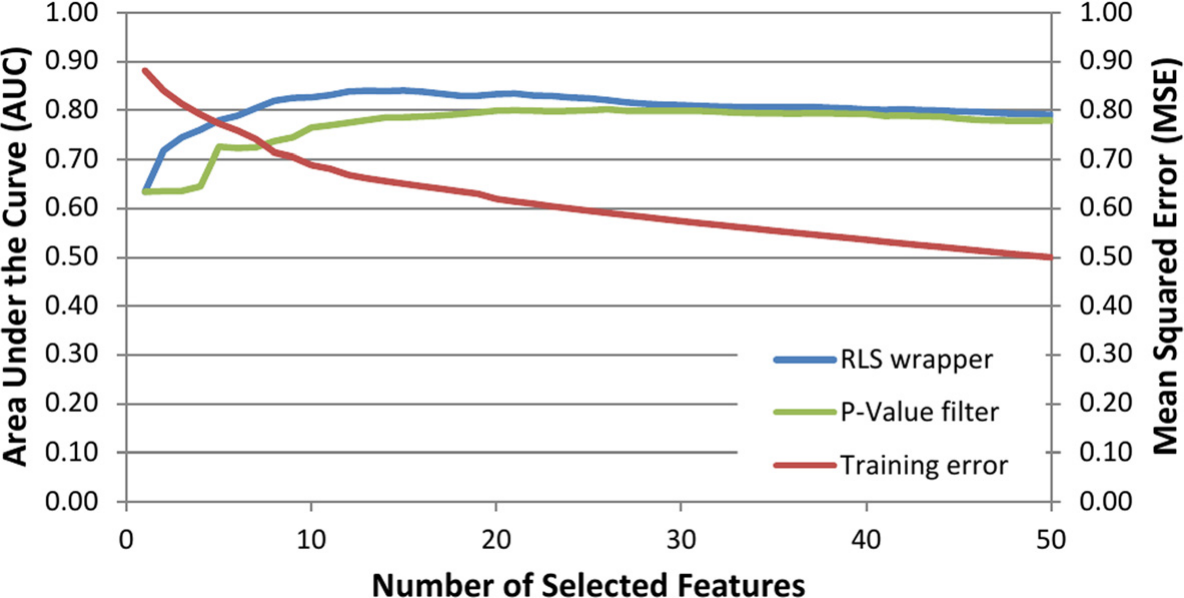
**The figure illustrates how the external and internal cross-validation results behave as functions of the number of selected features.** The external-cross validation consists of three training/test splits. The wrapper-based feature selection method, greedy RLS [23], is separately run during each round of the external cross-validation. Greedy RLS, in turn, employs an internal leave-one-out cross-validation on the training set for scoring the feature set candidates. The red curve depicts the mean values over these internal cross-validation errors. As can be easily observed from the blue curve, this internal cross-validation MSE used for the model training keeps constantly improving, which is expected, because the internal cross-validation quickly overfits to the training data when it is used as a selection measure. The blue curve depicts the area under curve (AUC) on the test data, held out during the external cross-validation round, that is, data completely unseen during the internal cross-validation and feature selection process. In contrast to the red curve, the blue curve starts to level off soon after the number of selected variants reaches around 10, indicating that adding extra features is not beneficial anymore even if the internal scoring function keeps improving. The green curve depicts the AUC of the RLS model trained using features selected by single-locus *p*-value based filter method, Fisher’s exact test, which is run with the same external training/test split as the greedy selection method. Similarly to the blue curve, the green one also stops improving soon after a relatively small set of features has been selected. The data used in the experiments is the Wellcome Trust Case Controls Consortium (WTCCC) Hypertension dataset combined with the UK National Blood Services’ controls.

One of the main challenges in feature selection is the accurate estimation of the prediction performance of the machine learning models on new samples unseen at the training phase, especially in settings in which the data is high-dimensional and the number of labeled training data is relatively small. Given the massive dimensionality of modern GWAS and NGS studies, it is in fact not very hard to find genetic features that can almost perfectly fit to a small training set but fail to generalize to unseen data, a phenomenon known as ***model overfitting***. Therefore, the models learned from genetic data should always be tested on independent data not used for training the model. In case the number of labeled data is small, one must resort to ***cross-validation*** techniques that repeatedly split the data into training and test sets, and the predictive accuracy is reported as an average over the test folds. In many applications of genomic predictors, there are a number of examples of the so-called ***selection bias***[40], meaning that the cross-validation is used to estimate the performance of the learning algorithm only, but not the preliminary feature selection done on the whole data, therefore leading to information leak and grossly over-optimistic results. Further, if cross-validation is used for selecting the hyper-parameters of the learning algorithm or for feature selection, this needs to be done within an internal cross-validation loop, separately during each round of an outer cross-validation loop [40-43]. This two-level technique is sometimes referred to as the ***nested cross-validation***[42,44]. An example demonstrating the behavior of a cross-validation error when it is used as a selection criterion with greedy forward selection is presented in Figure 1. The error curve that constantly decreases as a function of the number of selected features clearly indicates that the cross-validation becomes a part of the training algorithm itself in the inner loop, and therefore it cannot be trusted as a measure of true prediction performance for unseen data.

The evaluation of the predictive power is important also when considering predictive models constructed on the basis on statistical significant variants. For instance, there are numerous observations showing that the increases in the proportion of variance explained by significant variants does not go hand in hand with improved genetic prediction of disease risk. For instance, when using statistical modeling on the single training sample only, a panel of thousands of non-significant variants collectively could capture over one-third of the heritability for schizophrenia, but the same panel only explained a few percent of disease susceptibility in another replication cohort [8]. Similarly, while the statistical explanation power of the genetic variation in human height could be substantially increased by considering increasing number of common variants in a single population sample [45], the proportion of variance accounted for in other independent samples was much smaller [46]. These examples underscore the importance of rigid validation of the predictive accuracy of the models based on genetic profiles. While external cross-validation is a valid option, it is not free of any study-specific factors. For example, if there is a problem during the genotyping phase, it will appear also in any training and test data splits. These errors, stemming from problems during the experimental design and/or quality control have led for the need to re-evaluate the established methods and use caution when claiming replication [47]. The recommended option for truly validating the generalizability of predictive risk models is to make use of a large enough set of independent samples in which there is no overlap between the examined cohorts [48]. However, here one should consider whether the aim is to validate the predictive model itself (e.g. using external cross-validation or independent validation samples), or the predictive variants selected by the model (replication of the model construction or its application to separate cohorts) [49].

Through the development of better model validation techniques and unbiased examination of all feature subsets in genome-wide scale, we are likely to continuously improve the accuracy of the predictive models and increase their reproducibility on independent population samples. A challenge here is that differences in the population genetic structure, attributable to confounding factors such as the ethnicity or ancestry of the subjects, may result in highly heterogeneous datasets with a number of hidden subject sub-groups, which may associate with divergent disease phenotypes and therefore cause an increased false-positive rates [50]. Related to this, while there are comparisons among various feature selection methods and predictive modeling frameworks on individual cohorts [23,24,27], there is not yet any definitive results whether one method will universally lead to optimal results in other subject cohorts or populations. Such confounding variability should also be taken into account in the model construction and evaluation, perhaps in some form of population stratified cross-validation. Failure to replicate a genetic association should not only be considered as a negative result, as it may also provide important clues about genetic architecture among study populations or genetic interactions among risk variants [51]. When epistasis interactions are involved, then it is likely that simple methods, such as single-locus filters, will not alone be able to provide most optimal results, while in extremely large datasets, wrapper methods may pose computational limitations if combined with complex prediction models. Finally, even though the improvements obtained by the machine learning wrappers, compared to those from the traditional *p*-value based filters, may seem quite modest (e.g. Figure 1), it may turn out that even slight improvements in the predictive accuracy can result in significant clinical benefits. Moreover, it is argued that the modest predictive improvements may be further aggregated through pathway and network-level analyses of the selected variants.

## Molecular networks as a prior information for constructing predictive models

Even in the absence of significant single-locus marginal effects, multiple genetic loci from a number of molecular pathways may act synergistically and lead to disease phenotype when combined. Therefore, it has become popular to map the genetic loci identified in GWA or NGS studies to established biological pathways in order to elucidate the potential cellular mechanisms behind the observed genetic and phenotypic variation. There exist a wide variety of tools and guidelines on how to implement such pathway analyses in the context of genetic association studies [52-56]. Building on approaches originally developed in the context of microarray gene expression experiments, the common theme in the pathway analysis approaches is that they examine whether a group of related loci in the same biological pathway are jointly associated with a trait of interest. In line with the observations in microarray gene expression studies, it has been shown that in those cases where there is only a modest overlap in the variant or gene-level findings between different studies, due to factors such as differences in the genetic structure, the pathway-level associations may be much more reproducible even between different study populations [57-60]. These findings support the concept that individuals with the same disease phenotype may have marked inter-individual genetic heterogeneity in the sense that their disease predisposing variants may lie in distinct loci within the same or related pathways [14]. Machine learning-based predictive models constructed upon gene expression profiling have already shown the benefits of using pathway activities as features in terms of improved classification accuracy, compared to those models that consider merely individual gene expression levels [61]. It has also been demonstrated in the context of GWA datasets that pathway analysis can provide not only mechanistic insights but also improved discrimination power using tailored statistical data mining techniques, such as HyperLasso [62] or so-called pathways of distinction analysis (PoDA) [63].

**Figure 2:**
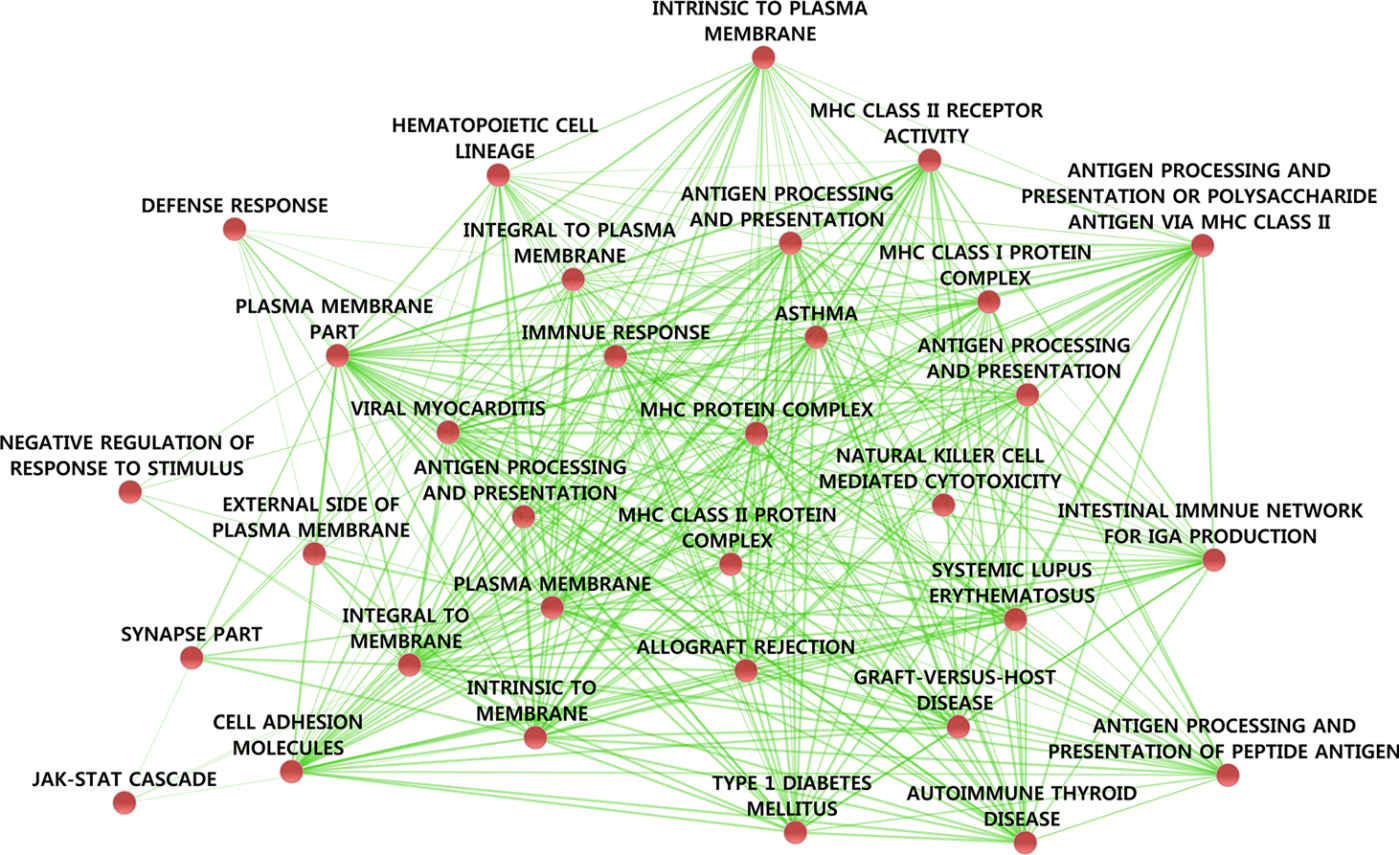
**Sample network visualization constructed for type 1 diabetes.** The risk variants were selected using the greedy RLS on the WTCCC type 1 diabetes GWAS data and the UK National Blood Services’ controls, extended with those genes selected in another work [62]. The biological processes and pathways were then mapped using DAVID [112,113], and the network visualization was done with the Enrichment Map plugin for Cytoscape [114,115]. The nodes represent pathways and the edges are the amount of overlap between the members of the pathways. The visualized network represents a selected sub-network of complex interconnections and cross-talks between a number of pathways, including MHC-related processes and other biological pathways associated with diabetes phenotypes. The pathways were identified initially using DAVID, with the criteria that they demonstrate enrichment when compared to the genome-wide background. The retrieved pathways were subsequently filtered in Cytoscape through the Enrichment Map plugin using the false-discovery rate and overlap coefficient to filter out non-significant pathways.

A limitation of constructing predictive models for a disease merely on the basis of established pathways is that these models may become biased toward already known biological processes, thereby potentially missing novel yet causal mechanisms predictive of the disease risk [64]. It may also not be so straightforward to infer the set of pathways that should be included in the model building process, in the absence of any *a priori* knowledge. Perhaps more importantly, statistical analysis of separate biological pathways or distinct gene sets undermines the effect of pathway cross-talk behind disease development, in which multiple genetic variants from distinct molecular pathways show synergistic contribution to the disease phenotype. In practice, the regulatory relationships behind many phenotypes are determined by complex and highly interconnected networks of physical and functional interplay between a multitude of pathway components [16]. As an example, we constructed a network representation for variants predictive of type 1 diabetes risk, which illustrate a selected portion of the number of pathways and their relationships that may be predictive of the disease onset (Figure 2). Given such high degree of interconnectivity, not only between the genetic variants but also among the implicated pathways, it is not surprising that the first machine learning frameworks for explicitly accounting epistatic gene-gene interactions have focused mostly on measures from information theory, such as those based on additive models, information gain, conditional entropy, or mutual information [24,65-67]. These models treat pairwise genetic interactions in a way that closely resembles the classic definition of epistasis, involving single and double-deletion experiments in model organisms [68]. However, even if allowing computationally efficient exploration of genetic interactions, *a posteriori* detection and heuristic search schemes cannot guarantee that the detected pairs of genetic risk factors will eventually be the most essential ones for the improved predictive power among all the possible variant combinations.

Toward more systematic network-centric analysis of genetic variants on a genome-wide scale, molecular interaction networks can be used as *a priori* information in the predictive models, in the form e.g. filters or integrators, with the aim of either reducing the massive size of the search space in the variant selection process or boosting the signal-to-noise ratio through external knowledge incorporated in terms of physical or functional molecular networks [69,70]. Network graphs provide a convenient mathematical framework for modeling, integrating and mining high-dimensional genomic datasets, in which to present the relationships among genetic loci, genes and diseases [64,69-72]. Successful examples of combining individual-level gene expression measurements with background networks of physical interactions between proteins and transcription factor targets have demonstrated that it is possible to identify and make use of disease-specific sub-networks, so-called ***modules***, in order to reduce both the number of false positives and negatives, caused by factors such as technical variability and genetic heterogeneity, respectively, as well as to improve individual-level prediction of clinical outcomes, such as cancer metastasis or survival time [64,73-75]. There are also studies in the context of GWA datasets, which motivate the use of network connectivity structures, such as sub-network modules or highly-connected network hubs [22,64,76-78], as aggregate features in the disease prediction models. However, what has been largely missing is a systematic approach that could combine network topology as *a priori* information when constructing predictive models. Recently, a particularly interesting approach was introduced as a principled method that uses genetic algorithms guided by the structure of a given gene interaction network to discover small groups of connected variants, which are jointly associated with a disease outcome on a genome-wide scale [79]. Combined with more efficient, wrapper-type of search algorithms, such network-guides feature selection approaches could be scaled-up in the future to enable extracting also larger sub-networks with improved predictive capability.

## Future directions: lessons from model organisms and individualized medicine

Given the rather modest progress made so far in pursuing the expensive and suboptimal route of current drug discovery, there has been much interest lately in moving towards ***personalized medicine*** strategies [80,81]. Another major paradigm shift in disease treatment is moving away from the traditional 'one target, one drug' strategy towards the so-called ***network pharmacology***, a novel paradigm which provides more global understanding of the mechanisms behind disease processed and drug action by considering drug targets in their context of biological networks and pathways [82]. These emerging paradigms can offer holistic information on disease networks and drug responses, with the aim of identifying more effective drug targets and their combinations tailored for individualized treatment strategies. A prime challenge in developing such strategies is to understand how genes function as interaction networks to carry out and regulate cellular processes, and how perturbations in these cellular networks cause certain phenotypes, such as human diseases, in some individuals, but not in the others. There has been active research in model organisms addressing the question why disease causing mutations do not cause the disease in all individuals [14]. Recent studies in yeast *Saccharomyces cerevisiae*, worm *Caenorhabditis elegans*, and fly *Drosophila melanogaster* have demonstrated the importance of incorporating functional genetic interaction partners of the mutated genes in the prediction of phenotypic variation and mutational outcomes at an individual level [83-85]. Pilot studies in human trials have also suggested that personal genomic approaches, such as those based on GWA or NGS studies, may indeed yield useful and clinically relevant information for individual patients [1,2]. However, a number of experimental, modeling and computational challenges have to be solved before the promises of personalized medicine can be translated into routine clinical practice [5,81,86].

From the experimental point of view, the whole-genome sequencing efforts will enable us to delve deeper into the individual genomes by elucidating the role of low-frequency variants in the genetic architecture of complex diseases. The sequencing efforts, such as the 1000 Genomes project [10], are also being used to subsequently extend the coverage of the existing GWA datasets by means of imputation methods and population-specific reference haplotypes [87,88]. However, while the emerging shift from population-level common variants toward individual-level rare or even personal variants holds great promise for medical research, it also represents with unique modeling challenges; in particular, the traditional statistical modeling frameworks that were developed under settings where the number of study samples greatly exceeds the number of study variables may not to be ideally suited for the personalized medicine settings, in which the individuals and disease subtypes are stratified into increasingly smaller subgroups [89]. Although machine learning methods are better targeted at individual-level prediction making, the feature selection methods would also benefit from more stratified options, for instance, in terms of enabling phenotype-specific genetic features, rather than assuming that all subjects share the same panel of predictive genotypes. Also, since the binary disease outcomes, typically in the form of case or control dichotomy, may not provide the most reliable study phenotypes, the predictive modeling frameworks might become more successful for predicting quantitative phenotypic traits [90-92]. This also raises related modeling questions, such as how to encode imputed variants (e.g. expected or most likely genotype), how to treat missing data (exclude or impute), or how to model the variants and their interactions (multiplicative, additive, recessive or dominant models) [90-94]; these all may have an important effect on the prediction performance, especially in the presence of epistatic interactions at an individual level.

From the computational perspective, the ever increasing sizes of the raw NGS and imputed GWA datasets pose great challenges to the computational algorithms. For instance, while systematic genetic mappings in model organisms have revealed widespread genetic interactions within individual species [85,95-97], epistasis interactions have remained extremely difficult to identify on a global scale in human populations. This can be attributed to the vast number of potential interaction partners, along with complex genotype-phenotype relationships and their individual-level differences. Improvements in computational performance have recently been obtained through effective usage of computer hardware, for instance, through graphics processing units, Cloud-based computing environments, or multithread parallelization, when exploring genetic variants or their interactions in GWA studies [98-101]. Furthermore, since the memory consumption in the high-dimensional NGS applications can form even a tighter bottleneck than the running time, there is also a need to develop space-efficient implementations, which trade running time for decreased memory consumption [23]. Lessons from model organisms, such as yeast, have also demonstrated that data integration between complementary screening approaches, either functional or physical assays, can reveal novel genetic interactions and their modular organization which have gone undetected by any of the individual approaches alone [95,96,102]. Also, integrating diverse phenotypic readouts facilitates genetic interaction screens [103], and Bayesian models have been shown especially useful for making use of multiple traits, gene-gene or gene-environment interactions in disease risk prediction [104]. Finally, visualization algorithms that can capture the hierarchical modularity of the physical and functional interaction networks may help reveal interesting biological patterns and relationships within the data, such as pathway components and biological processes, which can be further investigated by follow-up computational and/or experimental analyses [105].

Better understanding of the general design principles underlying genetic interaction networks in model organisms can provide important insights into the relationships between genotype and phenotype, toward better understanding and treating also complex human diseases, such as cancers. Cancer phenotypes are known to arise and develop from various genetic alterations, and therefore the same therapy often results in different treatment responses. Moreover, the underlying genetic heterogeneity results in alterations within multiple molecular pathways, which lead to various cancer phenotypes and make most tumors resistant to single agents. Cancer sequencing efforts, such as The Cancer Genome Atlas (TCGA), are systematically characterizing the structural basis of cancer, by identifying the genomic mutations associated with each cancer type. These efforts have revealed tremendous inter-individual mutational and phenotypic heterogeneity, which renders it difficult to translate the genetic information into clinically actionable individualized treatment strategies [106-108]. Therefore, integrating the structural genomic information with systematic functional assessment of genes for their contribution to genetic dependencies and cancer vulnerabilities, such as oncogenic addictions or synthetic lethalities [109,110], is likely needed for providing more comprehensive insight into the molecular mechanisms and pathways behind specific cancer types and for improving their prevention, diagnosis and treatment [106,111]. Machine learning-based predictive modeling approaches are well-powered to make the most of the exciting functional and genetic screens toward revealing hidden genetic variants and their interactions behind cancer and other complex phenotypes. When combined with network analyses, these integrated systems medicine approaches may offer the possibility to identify key players and their relationships responsible for multi-factorial behavior in disease networks, with many diagnostic, prognostic and pharmaceutical applications.

## Competing interests

The authors declare that they have no competing interests.

## Authors' contributions

SO contributed to the drafting of the manuscript and conducting experiments for the illustrations. TP contributed to the drafting of the manuscript. TA conceived the study, participated in the design of the experiments and contributed to the drafting of the manuscript. All authors read and approved the final manuscript.
